# Lentivirus-mediated shRNA interference of clusterin blocks proliferation, motility, invasion and cell cycle in the ovarian cancer cells

**DOI:** 10.1186/s13048-015-0173-z

**Published:** 2015-08-21

**Authors:** Yanxia Fu, Yingrong Lai, Junfeng Liu, Xingyang Liu, Zeshan You, Guofen Yang

**Affiliations:** Department of Gynecology, The First Affiliated Hospital of Sun Yat-Sen University, Guangzhou, China; Department of Pathology, The First Affiliated Hospital of Sun Yat-Sen University, Guangzhou, 510080 Guangdong China

**Keywords:** Ovarian cancer, Clustein

## Abstract

**Background:**

In a previous analysis on the patients with ovarian cancers, we have found that clusterin is a biomarker associated with ovarian cancer *in vivo* and may be a prognostic factor associated with adverse outcome. Here, we explored the effect of lentivirus-mediated shRNA interference of clusterin, investigated whether clusterin was associated with adverse outcome of ovarian cancer cells *in vitro*.

**Methods:**

OVCAR-3 and TOV-21G cell lines were infected with the lentivirus for delivering clusterin shRNA, and the stably transfected cells were selected. The effect of clusterin silencing was detected by western blotting assay. The proliferation, clonability, migration, invasion and cell cycle of two cell lines were detected separately by MTT assay, clone formation assay, scratch assay, transwell assay and fluorescence-activated cell sorting.

**Results:**

Following clusterin silencing with shRNA, the expression of clusterin in two cell lines were decreased. And the proliferation, clonability, migration, invasion of these two cell lines were down-regulated apparently. The cell cycle of two cell lines was disturbed, cells in G1 phase was increased, but cells in G2 and S phase was decreased.

**Conclusions:**

The expression of clusterin is significantly correlated with the biological characteristics of ovarian cancer cells, it may be a potential molecular for ovarian cancer treatment.

## Introduction

Ovarian cancer, a major cause of death in all gynecologic malignancies, is one of the most common malignancy in women worldwide and is the fourth leading cause of death from malignant disease [1]. Recently, the incidence of ovarian cancer has been increasing in China, Singapore and other countries [2]. With optimal surgical treatment and standard chemotherapy, the 5-year survival rate of ovarian cancer patients has improved significantly [3]. Nevertheless, the preliminary success in tumor regression and recurrence can’t prevent resistance to further chemotherapeutic treatment. Development of this further resistance suggests the major limitation to the treatment. Hence, there is a crying need to look for a suitable solution for further treatment.

Recently, the molecular targeted drug and treatment is widely used in tumor treatment. Lots of reports show that there are many specific genes which related to tumor genesis and development of the ovarian cancer, such as CIP2A [4], HOX [5], BRIP1 [6], EIF5A2 [7] and Bmi-1 [8]. Our previous studies suggested clusterin was probably another gene relevant to the treatment of ovarian cancer.

Clustein is a highly conserved, heterodimeric-secreted glycoprotein, which is encoded by a single copy gene located on chromosome 8p21-p12. Clustein is known as apolipoprotein J, estosterone-repressed prostate message-2, or sulfated glycoprotein-2. Clusterin gene expression is complex, appearing as different forms in different cell compartments [9]. Recent studies indicated that clusterin gene expressed in kinds of human tumor cells, including liver cancer cells, prostate cancer cells, colon cancer cells and bladder cancer cells [10–13]. The clusterin expression was up-regulated in all these cells, and the level of expression was closely related to the tumorigenesis of the tumor.

Previous research reveal clusterin was up-regulated in the ovarian cancer cells and the overexpression of clusterin did affect the tumorigenesis of the tumor [14]. To further study the function of clusterin, we silented the clusterin gene of ovarian cancer cells with the lentivirus vector *in vitro* and detected the clusterin gene expression in silenced-tumor cells. Moreover, we assessed the proliferation, migration, invasion, clonability, cell cycle of ovarian carcinoma cells after clusterin gene silencing.

## Materials and methods

### Cell line and culture conditions

Human ovarian cancer cell lines OVCAR-3 and TOV-21G were obtained from Sun Yat-Sen University Cancer Center (Guangzhou, China). Human ovarian cancer cell lines HO8910 and HO8910PM were purchased from Shanghai cell bank of Chinese academy of sciences. OVCAR-3, HO8910 and HO8910PM cells were growth in RPMI1640 mediumwith 10 % (v/v) fetal calf serum, streptomycin (100 U/ml) and penicillin (100 U/ml). TOV-21G were growth in MCDB105, Medium199 mixed Medium (1:1) with 10 % (v/v) fetal calf serum, streptomycin (100 U/ml) and penicillin (100 U/ml). RPMI1640 medium, fetal bovine serum (FBS) and Dimethylsulfoxide (DMSO) were purchased from Gibco Biotechnology (Gibco-BRL, MD, USA). MCDB105, Medium199 were purchased from Sigma (USA). Cultures were maintained at 37 °C in an incubator with a humidified atmosphere of 5 % CO_2_.

### Western blotting to analyze the clusterin gene expression in tumor cells

For western blotting analysis, cells were seeded in 6-well plates at 2×10^5^/well. Cells were grown to 90 % confluence and were lysed in cell Lysis solution (RIPA: PMSF = 100:1) for 30 min and were transferred to 1.5 ml EP for 30 min on ice. Lysates were centrifuged at 12000 g for 30 min to remove nuclei and precipitates. Supernatant protein concentrations were measured using the Bio-Rad protein assay (OD:562 nm) with BSA in lysis buffer as a standard. Cell lysates were loaded into each well containing SDS-PAGE and transferred to nitrocellulose membranes. The protein concentration were adjusted to 40 μl. Membranes were blocked for 2 h at room temperature in 0.1 % TBS with 5 % non-fat milk, and probed using Clusterin antibody (1:100) purchased from Millipore (Billerica, MA, USA) andα-tubulin (1:1000) as the internal control purchased from (Santa Cruz, CA, USA) overnight. After the membrane washing three times by 0.1 % TBS, the secondary antibody was added and incubated 2 h at room temperature. Then the bands were visualized by an ECL kit (ThermoScientific Pierce).

### Lentivirus constructions

ShRNA was designed by Shanghai Jikai gene Chemical Co., Ltd. (Shanghai, China) and referred to Clusterin Gene (NM_203339) of GeneBank. The PGCSIL-GFP, which is a third generation self-inactivating lentivirus vector containing a CMV-driven GFP reporter and a U6 promoter upstream of cloning restriction sites, was used in the shRNA silencing system. The synthetic oligonucleotide primers used were CLU; forward (5’- CCGGGACCAGACGGTCTCAGACAATCTCGAGATTGTCTGAGACCGTCTGGTCTTTTTG-3’) and reverse (5’-AATTCAAAAAGACCAGACGGTCTCAGACAATCTCGAGATTGTCTGAGACCGTCTGGTC-3’). The primers were annealed and linked into the cloning restriction site of the vector which had been digested with the restriction enzymes AgeI and EcoRI. After annealing, the double-stranded DNA was digested with EcoRI to linearize the pGCSIL-GFP vector. The negative control sequence (5’-ttctccgaac gtgtcacgt-3’) was used as previously described. The NC-shRNA was designed; forward forward (5’-ccggaaccagagctcgcccttctacttcaagagagtagaagggcgagctctggtttttttg-3’) and reverse (5’-aattcaaaaaaaccagagctcgcccttctactctcttgaagtagaagggcgagctctggtt-3’). It has been proven to be efficient in Clusterin silencing experiments. Then it was co-transfected with pHelper 1.0 and pHelper 2.0 into 293T cells to package and produce the shRNA expressing lentivirus. The supernatant was collected and concentrated 48 h after co-transfection. The titer of lentivirus targeting Clusterin (LV-CLU) and lentivirus targeting negative control (LV-NC) was examined by the hole by dilution titer method. The vectors and oligonuleotide primers were purchased from Genechem. To knock down the Clusterin in the OVCAR-3 and TOV-21G cancer cell lines, cells were seeded in a 6-well tissue culture plate with 2×105/well 1 day prior to infection. The complete culturesolution was replaced by infection enhancing solution with 5 μg/ml polybrene (Genechem) and the packed lentivirus was added to cells with multiplicity of infection (MOI) 20 or 10. Twelve hours later, the lentivirus solution was replaced with complete culture solution. Infected cells were subcultured every 5–7 days [13].

### Test the infection and knockdown efficiency

The human tumor cells grew well on the day prior to viral introduction was recovered, and were incubated with 5 % CO_2_ at 37 °C. Following the incubation, the expression of GFP was observed under a fluorescence microscope. When the efficiency of infection exceeded 50 %, Cells were collected. The protein expression of clusterin gene were analyzed using western blotting as above.

### MTT assay and clone formation assay to detect the proliferation of ovarian cancer cells

Cells were cultured in the 6-well plates at 2×10^5^/well. When cells were grown to 80 % confluence, they were trypsinized. The cell suspension was re-suspended in complete medium. Cells were counted and added in 96-well plate at 1000 cells/well. Each group, included three compound perforations, added 100μl complete medium. Cells were incubated with 5 % CO_2_, 37 °C. The growth of cells were tested with Enzyme immunoassay for four days. The data were collected and analyzed to create a proliferation curve.

Meanwhile, the suspending cells were transferred into six-well plate at 300 cells/well. Each well was added 2 ml complete medium and comprised three compound perforations. Cells were incubated with 5 % CO_2_ and cultured at 37 °C until the cell clones could be observed directly. The medium was removed, methanol was added for 30 min. Removing methanol, the clones were dyed with crystal violet for 30 min and counted.

### Scratch assay and transwell assay to detect the migration and invasion of ovarian cancer cells

Cells were cultured six-well plate at 5 × 10^5^ cells/well. Each well was added 2 ml complete medium with 10 % PFA and comprised three compound perforations. Cells were incubated with 5 % CO_2_ and cultured at 37 °C. When cells were covered almost confluent, the surface of cells was scratched by the same pin and washed twice with PBS, allowed to migrate in RPMI1640 without FBS for 24 h. Phase contrast micrograph images were captured immediately and 24 h later. The relative distance traveled by the leading edge from 0 to 22 h was assessed using Adobe Photoshop CS3 software (Adobe Inc.) (*n* = 6).

Cells were transferred into 24-well plate at 5 × 10^4^ cells/well and cultured as above for 24 h. After trypsinized, cells washed with PBS and resuspended in RPMI-1640 without FBS. Samples of 5 × 10^4^ cells were placed in the upper chamber of each Transwell device (Falcon, BD Labware, Bedford, MA, USA) with an 8-μm Matrigel-coated polycarbonate membrane filter inserted in 24-well plates. RPMI-1640 with 10 % FBS was placed in the lower chamber. After 24 h of incubation, the non-invading cells were removed by wiping the upper surface of the filter with a cotton swab. The remaining cells were fixed in 100 % methanol for 20 min, stained with Giemsa (Sigma), and rinsed with distilled water several times. The degree of invasion was quantified by selecting five different predetermined views (original magnification, x200) and counting the cells on the underside of the filters under a microscope [13].

### Fluorescence-activated cell sorting (FACS) to assess ovarian cancer cell cycle distribution following clusterin gene silencing

Cells were cultured six-well plate at 5 × 10^5^ cells/well. The cell supernatant was collected when the coverage rate in the experimental group increased to 80 %, ensuring that cells were in the logarithmic phase. Cells were washed twice with PBS and subjected to trypsinization. Cells were collected in a 5 ml centrifuge tube. Three compound perforations were prepared in each group and timed cycle tests were performed ensuring an adequate number of cells for computerized analysis, with at least 1,000,000 each time. Cells were collected after centrifugation at 2,000 rpm for 5 min and fixed with 70 % ethanol, which was pre-cooled to 4 °C overnight. The stationary liquid was abandoned by centrifugation at 2000 rpm for 5 min. Cells were washed with PBS twice. Cells were incubated in PBS containing 400 μl RNase (50 μg/ml) at 37 °C for 30 min. Cells were then stained with 800 μl PI (20 μg/ml) for 30 min in the dark at 37 °C. Cell cycle progression was analyzed using a flow cytometer (Beckman Coulter, Miami, FL, USA). During cell cycle analysis, gating and voltage were carefully set to exclude clumped cells and cell debris. The data were analyzed using CXP Analysis 2.0 (Beckman Coulter).

### Statistical analysis

All data are analyzed with the statistical software SPSS 16.0 and expressed as mean ± standard deviation (SD). The significance level of statistics was set at *P* < 0.05.

## Results

### The expression of clusterin protein in tumor cells

**Fig. 1:**
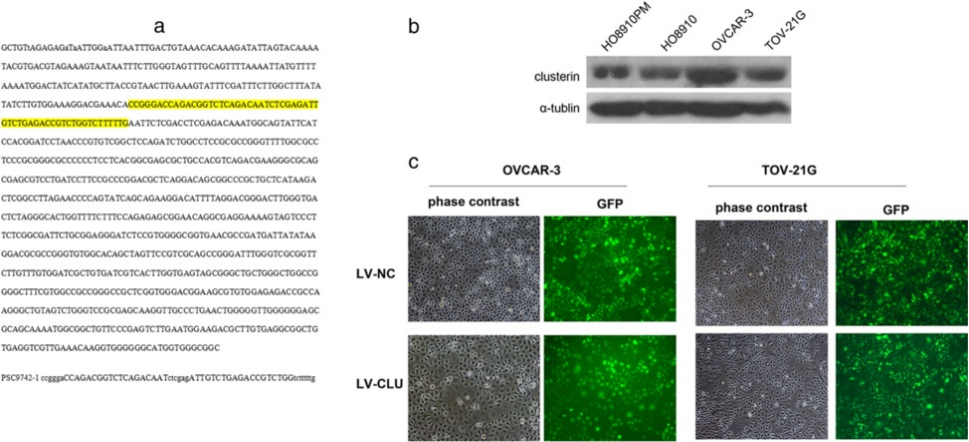
Establishment of stable CLU silencing OVCAR-3 and TOV-21G cell lines. **a** Detection of lentivirus transfection in OVCAR-3 and TOV-21G cells. The results of gene sequencing showed that OVCAR-3 and TOV-21G cells were transfected successfully LV-CLU or LV-NC gene with lentivirus (**b**) Analysis of levels of Clusterin protein in OVCAR-3 and TOV-21G cells. Results suggested that both OVCAR-3 and TOV-21G cells had the higher level of Clusterin expression. **c** Clusterin (GFP) expression in OVCAR-3 and TOV-21G cells as detected by fluorescence microscope (x100). The infection efficiency of OVCAR-3 cells (MOI 20) and TOV-21G cells (MOI 10) were shown. The cells were infected with LV-CLU or LV-NC. Four days later, the infection efficiency achieved was >90 %

To select the cell lines with a high level of CLU expression, we used western blot analysis to analyze the Clusterin expression at the protein levels in HO8910, HO8910PM, OVCAR-3 and TOV-21G cancer cell lines. The results of the western blotting, which set α-tublin as an internal reference, suggested that the clusterin protein was separately expressed in the HO8910, HO8910PM, OVCAR-3 and TOV-21G cells (Fig. 1b). The levels of clusterin protein were higher in the OVCAR-3 and TOV-21G cells than in the HO8910 and HO8910PM cells. It was found that the protein expression level of two cell lines was different, but was abundant.

**Fig. 2:**
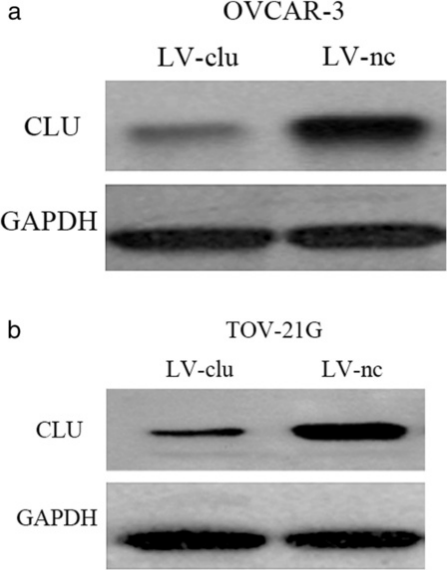
Clusterin protein expression in OVCAR-3 and TOV-21G cells following knockdown of the clusterin gene. **a** the protein expression of clusterin in the OVCAR-3 cells. The western blot analysis demonstrated Clusterin protein expression reduced in LV-CLU group, while Clusterin protein expression was almost unaffected by the LV-NC. **b** the protein expression of clusterin in the TOV-21G cells. The results was the same as the OVCAR-3 cells

Transfected with the shRNA lentivirus, GFP expression was observed under fluorescence microscopy (Fig. 1c). The infection efficiency of OVCAR-3 cells (MOI 20) and TOV-21G cells (MOI 10) were shown. The cells were infected with LV-CLU or LV-NC. Four days later, the infection efficiency achieved was >90 %. The level of GFP expression reached respectively the highest levels on the fourth day. To further, the gene sequence of the OVCAR-3 and TOV-21G cells were sequenced (Fig. 1a). The levels of CLU protein in the OVCAR-3 and TOV-21G cells were detected by western blotting. The results showed that the levels of CLU protein were significantly lowered in cells of LV-CLU groups. However, there was no significantly influence in the NC-CLU groups (Fig. 2).

### Analysis of inhibition of the proliferation of ovarian cancer cells following down-regulation of the clusterin gene

**Fig. 3:**
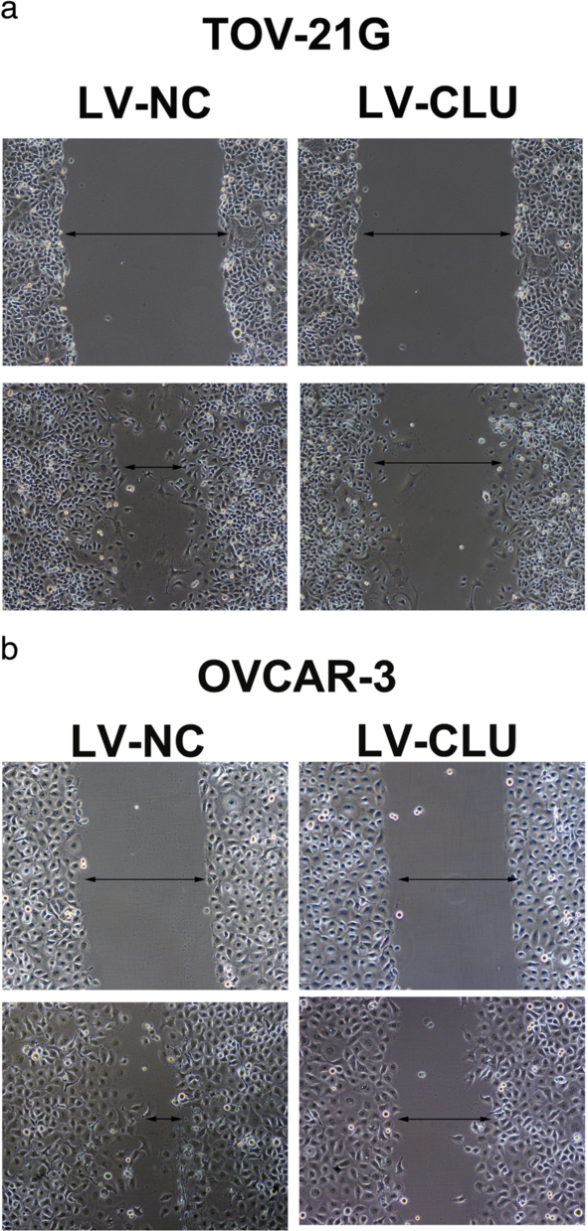
The results of the migration following Clusterin gene silencing (**a**) A scratch assay was used to detect the migration ability of TOV-21G cells after Clusterin silencing. The two groups were treated with a pin, respectively, for 24h. The cells of LV-CLU group had a greater migrating distance compared to the cells of NC-CLU group. (**b**) A scratch assay was used to detect the migration ability of OVCAR-3 cells after Clusterin silencing. The results was similar to the TOV-21G cells

The proliferation of ovarian cancer cells were detected by MTT assay. After transfection with the shRNA lentivirus, the proliferation rate of OVCAR-3 cells in LV-CLU group was inhibited together with the NC-CLU control from the third day (Fig. 3). And compared with the NC-CLU control, the proliferation rate of TOV-21G cells in the LV-CLU group was inhibited from the second day (Fig. 3). The results show that down-regulation of the clusterin gene in two cell lines were obvious.

**Fig. 4:**
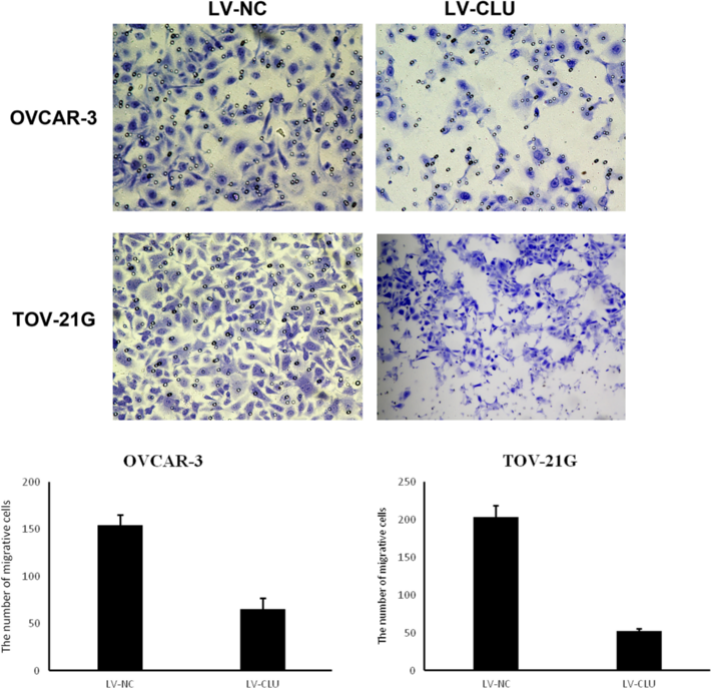
The results of the invasion following clusterin gene silencing. Statistical analysis revealed a significant difference between the LV-NC and LV-CLU groups. The results demonstrated that the cells of LV-CLU groups had less invasiveness

### Analysis of inhibition of the migration following clusterin gene silencing

The migration of tumor cells were detected by scratch assay and observed under microscopes. The two groups were treated with a pin, respectively, for 24 h. The cells of LV-CLU group had a greater migrating distance compared to the cells of NC-CLU group after 24 h. The results was similar to the TOV-21G cells (Fig. 3). This results suggest that the migration of OVCAR-3 and TOV-21G cells after the transfection was diminished.

**Fig. 5:**
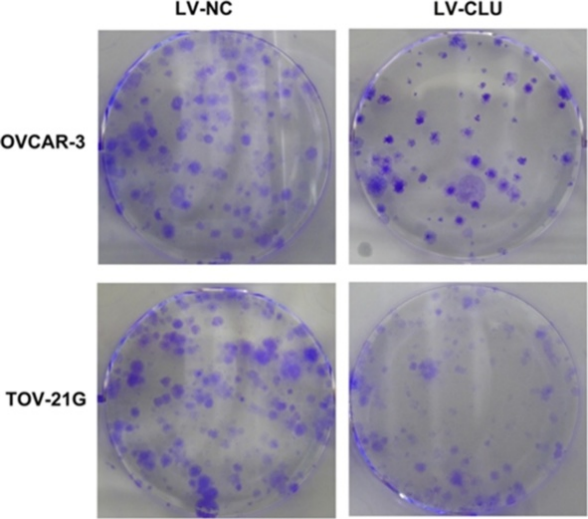
A plate clone formation assay is shown. The data demonstrated that OVCAR-3 and TOV-21G cells of LV-CLU group had a lower clone formation rate than the NC-CLU groups (P<0.05) (n=3)

### Analysis of inhibition of the invasion following clusterin gene silencing

Invasion is an important aspect that lead to the ability of cancer cells to metastasize. In our study, transwell assay was used to detect whether Clusterin silensing would affect cell invasion. Cells of LV-NC and LV-CLU groups were seeded in transwell chambers and cultured for 24 h. The results demonstrated that cells of LV-CLU group had less invasiveness than cells of LV-CLU group. Statistical analysis revealed a significant difference between the LV-NC and LV-CLU groups (*P* < 0.01) (Fig. 4).

**Fig. 6:**
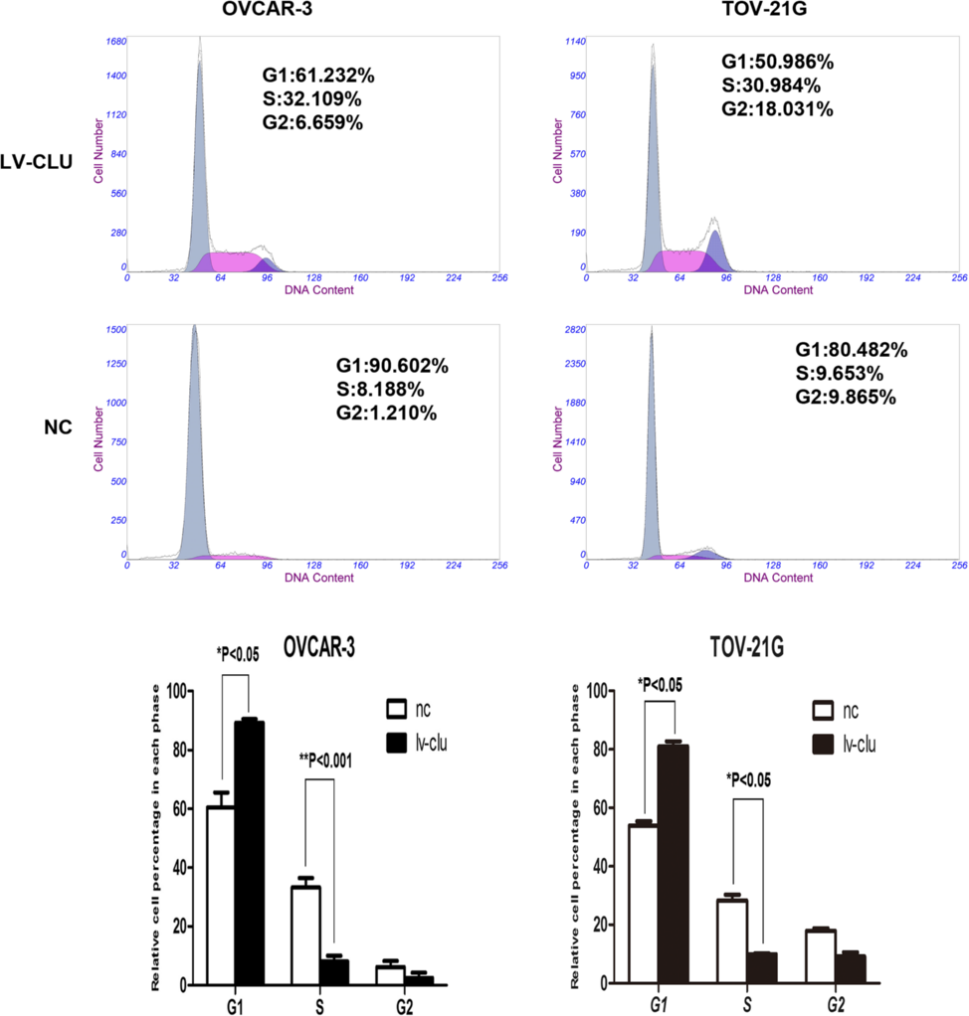
Changes in the cell cycle distribution of OVCAR-3 and TOV-21G cells in the knockdown group. After transfection with the shRNA lentivirus, the percentage of OVCAR-3 and TOV-21G cell cells in the G1 phase was significantly increased (P<0.05), while cells in the S (P<0.001) and G2 phases decreased significantly (P<0.05) in the LV-CLU group compared with the negative control

### Analysis of inhibition of the clonability following clusterin gene silencing

We observed a decrease in the number of OVCAR-3 and TOV-21G cell colonies in the LV-CLU group after clusterin gene silencing. The number of cells in the colonies decreased relative to the NC-CLU control (*P* < 0.05) (*n* = 3) (Fig. 5). The results indicates that the clonability of OVCAR-3 and TOV-21G cells largely inhibited following the down-regulation of clusterin gene.

### FACS analysis of cell cycle following clusterin gene silencing

After transfection with the shRNA lentivirus, the percentage of OVCAR-3 and TOV-21G cell cells in the G1 phase was significantly increased (*P* < 0.05), while cells in the S (*P* < 0.001) and G2 phases decreased significantly (*P* < 0.05) in the LV-CLU group compared with the NC-CLU control (Fig. 6). The results indicates that silence of the clusterin gene was apparently related to regular distribution of the OVCAR-3 and TOV-21G cells.

## Discussion

In recently reports, the expression level of clusterin was studied in normal ovaries, benign and borderline ovarian tumors, and malignant epithelial cancers. A significant increasing cytoplasmic expression of clusterin was observed from the normal ovary, to benign cystadenoma, borderline tumor, and malignant epithelial carcinoma. These evidences suggest up-regulation of clusterin is associated closely with tumor progression [15]. The level of clusterin expression maybe is an important prognostic factor in the assessment of the aggressive nature of ovarian cancer [2].

Clusterin is an controversial molecule and it remains debatable about whether clusterin has multiple functions or only a single primary function influenced by cellular context [16]. It is not clear why clusterin play contradictory functions, such as cell survival, tumor progression, treatment resistance or cell apoptosis [17]. However, its functions in many cancer cells are certain. The level of clusterin expression in renal cancer cells was found to be closely associated with pathological stage and grade of the tumor, and the overall survival rate of patients with high clusterin expression was obviously lower than that of patients with weak expression [18]. The level of clusterin expression correlated with the level of estrogen and progesterone receptor expression, tumor size, and lymph node metastasis in breast carcinoma [19]. In colon carcinoma, clusterin has been used as a novel prognostic and predictive marker which is observed in aggressive tumors and metastatic nodules [20]. Similarly, clusterin expression is associated with FIGO stage and histological type in ovarian cancer [21]. Hence, through mechanisms not yet elucidated, it is certain that CLU biosynthesis is altered and up-regulated in cancer tissues.

In the preset study, we have showed that clusterin express highly in two ovarian cancer cell lines *in vitro*, while there is lower clusterin expression in the normal ovarian cells [14]. And the clusterin expression could be down-regulated with a lentiviral vector. Following the clusterin silencing, the clusterin expression was down-regulated, and the proliferation, clonability of OVCAR-3 and TOV-21G cells were decreased, meanwhile, the migration and invasion of these cells also down-regulated. The cell cycle of these cells were changed. A large number of OVCAR-3 and TOV-21G cells were confined in G1 phase, only a small number of these cells accessed into G2 phase and S phase. In contrast, no-silencing cells had a strong clusterin expression. They had significantly higher abilities of proliferation, clonability, migration and invasion. Our data suggest that clusterin is an important protein associated with both cancer therapy and tumor development.

Our results also support that clusterin is a stress-associated anti-apoptotic protein that is up-regulated in an adaptive cell survival manner following various cell death trigger in ovarian cancer cells [22, 23]. More evidence suggests that clusterin enhance the resistance to cytotoxic chemotherapy and radiotherapy in breast cancer [24], lung cancer, cervical cancer, and prostate cancer. Therefore, silencing clusterin expression to overcome chemoresistance maybe is an novel therapeutic strategy.

## Conclusions

In summary, present study demonstrated that the biogenesis of ovarian cancer cells was changed following the silence of clusterin. And the proliferation, clonability, migration, invasion and cell cycle of ovarian cancer cells are significantly correlated with the expression of clusterin. Therefore, clusterin could be a potential molecular for ovarian cancer treatment. Our study may be helpful to the development of new therapeutic regimens and improvement the subsequent survival in ovarian cancer patients.

## Abbreviations

shRNA: Short hairpin RNA
GFP: Green fluorescent protein
LV-CLU: Lentivirus targeting Clusterin
LV-NC: Lentivirus targeting negative control
